# An incidental finding during a brain plasticity study: substantial telomere length shortening after COVID-19 lockdown in the older population

**DOI:** 10.1007/s11357-025-01602-z

**Published:** 2025-03-18

**Authors:** Kirsten Jahn, Shambhabi Chatterjee, Christopher Sinke, Jonas Janik Ralf Koberschinski, Kristin Jünemann, Clara Eline James, Florian Worschech, Damien Marie, Eckart Altenmüller, Christian Bär, Tillmann Horst Christoph Krüger

**Affiliations:** 1 Laboratory of Molecular Neurosciences (LMN), Dept. Of Clinical Psychiatry, Hannover Medical School, Hannover, Germany; 2 Institute for Molecular and Translational Therapeutic Strategies (IMTTS), Hannover Medical School, Hannover, Germany; 3 Fraunhofer Institute for Toxicology and Experimental Medicine, Hannover, Germany; 4 Division of Clinical Psychology and Sexual Medicine, Dept. Of Psychiatry, Social Psychiatry and Psychotherapy, Hannover Medical School, Hannover, Germany; 5 Geneva Musical Minds Lab, Geneva School of Health Sciences, University of Applied Sciences and Arts Western Switzerland (HES-SO), Geneva, Switzerland; 6 Faculty of Psychology and Educational Sciences, University of Geneva, Geneva, Switzerland; 7 Institute of Music Physiology and Musicians′ Medicine, Drama and Media, Hannover University of Music, Drama and Media, Hannover, Germany; 8 Center for Systems Neuroscience, Hannover, Germany; 9 CIBM Center for Biomedical Imaging, Cognitive and Affective Neuroimaging Section, University of Geneva, Geneva, Switzerland

**Keywords:** Telomere length, Older population, Lockdown

## Abstract

The detrimental effects of lockdowns have already been proven by numerous studies, mainly using psychometric measurements. Since telomere shortening is a driver of aging and aging-associated disorders, including cognitive decline, the telomere length in the older population has been investigated in the current study. Measurements were taken over a 6-month period just before and during the 6 months that included the first lockdown. The cohort of 55 persons aged 64 to 70 years was investigated in the context of a study focusing on neuroplasticity. Participants were recruited in Germany and Switzerland and characterized by psychometric measurements concerning neurocognition and neuroplasticity. Telomere lengths were measured by real-time PCR-based LTL measurement. We found an impressive and significant decline in telomere lengths in the period that included the lockdown (2.33 (± 0.1) at T1 vs. 1.35 (± 0.1) at T2), whereas it was stable in the phase before the lockdown in the same individuals (T0 was 2.25 (± 0.1 S.E.M.) vs. T1, 2.33 (± 0.1)). Correlation of the sudden decrease revealed no linkage to health issues or general physical activity but was in trend related to a decline in the WHOQOL-BREF Social Score referring to the social interaction of the study participants. Our data support, at a biological level, the results of clinical and psychosocial studies showing the detrimental effects of lockdowns.

## Introduction

At the beginning of the year 2020, COVID-19 (coronavirus disease caused by SARS-CoV-2) reached Germany and the first measures were taken to prevent hospitals, already overstretched by cost cuts, from being overwhelmed. Therefore, there was a first lockdown from mid-March 2020 until mid-May 2020, after which a slight loosening of lockdown measures started. In Switzerland, there was a lockdown in the same period. Around this time (in 2019/2020), a project entitled “Train the Brain with Music” (TBM) studying the effect of musical training (piano lessons) on brain plasticity and aging in the older population was being held simultaneously at our medical centers in Hannover, Germany, and Geneva, Switzerland.

Replicative telomere shortening occurs throughout the lifespan of an organism and is a predictor of lifespan as the telomerase enzyme is shut down after birth [1]. Telomere attrition is strongly associated with mental disorders such as anxiety [2] and age-related disorders including neurodegenerative diseases [3, 4]. Researchers have also shown that telomerase therapy can help to curb a few symptoms of neurodegeneration highlighting the importance of telomere homeostasis for brain function [5]. Therefore, the telomere lengths were determined at three defined time points to investigate whether the music intervention could slow down the shortening of telomeres which is used as a marker for aging [6].

However, these time points incidentally framed a 6-month phase that preceded the first lockdown and a 6-month phase that included the first lockdown in most participants: the first time-point (T0) was mid-2019, the second time point (T1) was in the end (November/December) of 2019, and the third assessment (T2) was in the middle of 2020 (May/June). For safety reasons (to prevent the spread of the virus in the institutes), study subjects were asked on the third time point whether they had already been exposed to an infection with SARS-CoV-2. However, as infections were still rare in Germany at that time, none of the participants had been through an infection with SARS-CoV-2.

## Methods

### Subjects

Study participants were recruited at Hannover Medical School, Dept. for Clinical Psychiatry, Social Psychiatry and Psychotherapy, Division of Clinical Psychology and Sexual Medicine, and at the Geneva Musical Minds Lab, Geneva School of Health Sciences, University of Applied Sciences and Arts Western Switzerland (HES-SO), Geneva, Switzerland, via newspaper advertisement and placards at public places. Demographics are given in the results part in Table 2. Prerequisites for participation in the study were overall good health, absence of psychiatric diseases, being right-handed, retired, and non-reliant on hearing aids.

### Study design

The first time point for data assessment was before the weekly practical piano lessons were started (or theoretical music lessons without any practical exercises in the control group, respectively), the second time point was after half a year, and the third time point was 1 year after the first assessment (for the long-term observation of potential beneficial effects of piano-lessons) for every individual participant. The time points each cover a period of several months, as all the participants could not be examined on the same day, since MRI scans were also performed as part of the original study (T0: March to June 2019, T1: August 2019 to February 2020, T2: June 2020 to November 2020). A small subgroup of participants were recruited as early as in January 2019 (T0), and had their consecutive examinations in August 2019 (T1) and in the end of February/beginning of March 2020 (T2). In other words, their T2 was directly before the lockdown started. Therefore, this small subgroup was excluded from the main analyses. However, 55 patients were finally included in the present manuscript.

### Extent of COVID-19 lockdown

In both countries, Germany and Switzerland, lockdown measures were similar. It was forbidden to meet more than one other person except for the nuclear family. Major events were canceled as well as private marriages, and church services. It was not allowed to travel for vacation. Restaurants, cafes, bars, and discos were closed as well as barber shops, nail salons, and physiotherapy practices. Concerning retail, only supermarkets were kept open to ensure supplying of the population. Many companies stopped operating. Schools, most kindergartens, universities, libraries, swimming baths, and gyms closed down as well as all cultural facilities. Even playgrounds had been locked. Children were not allowed to meet other children and had to learn at home as school teaching was canceled for months. Most adults had to work in home office and for families, the situation at home became very dense. Furthermore, it was forbidden to visit hospitalized relatives or friends. Also, there was no possibility to visit old people in rest homes. Therefore, many old or sick people had to die lonely without any support from the ones they loved.

### Measurement of telomere lengths

#### DNA isolation

Genomic DNA (gDNA) for telomere measurements was isolated in the Institute of Molecular and Translational Therapeutic Strategies of Hannover Medical School according to standard procedures from 50 µL blood using the DNeasy Blood & Tissue Kit (Qiagen #69,506) and stored at − 20 °C. DNA samples were diluted in 96-well plates to a fixed concentration of 10 ng/µL. The blood samples were received anonymized and prior to gDNA isolation, the order of the samples was further randomized to minimize potential batch effects.

#### Real-time PCR-based LTL measurement

A quantitative polymerase chain reaction (qPCR)-based assay was performed to measure the relative telomere length as described before [7]. The ratio of the mean telomere (T) repeat sequence to a reference single copy (S) gene is calculated for each sample as previously described [8].

DNA samples were amplified in a total reaction volume of 10 µL containing 1 × iQ SYBR Green Supermix (Biorad #172-5006CUST), 2 µM ROX reference dye (Thermofischer #AB-4136/B) with either HPLC grade primer pairs for T or S amplification (see Table 1 below), and 20 ng of gDNA. For telomere repeat amplification, 300 nM of Human T FW primer and 900 nM of Human T RV primer were used. Whereas for S gene—36B4 primers were used for amplification for which the concentration of primers were as follows: 300 nM of 36B4 FW and 500 nM of 36B4 RV.

**Table 1:** Primers used in qPCR

Human T FW	5′- GGTTTTTGAGGGTGAGGGTGAGGGTGAGGGTGAGGGT-3′
Human T RV	5′-TCCCGACTATCCCTATCCCTATCCCTATCCCTACCCTA-3′
Human S FW	5′-CAGCAAGTGGGAAGGTGTAATCC-3′
Human S RV	5′-CCCATTCTATCATCAACGGGTACAA-3′

All DNA samples were run as triplicates on the qPCR (ABI Viia 7 Real-Time PCR system) for both the telomere (T) primer and 36B4 (S) primer, in 384-well format. The distribution of samples on the plates was randomized. The thermal cycling profile was the same for both primers: 95 °C incubation for 10 min followed by 35 cycles of 95 °C for 15 s, 54 °C for 2 min, and 72 °C for 15 s. Inter-run calibrators were included in each qPCR run comprising one gDNA sample with long telomeres (human-induced pluripotent stem cells) and another with short telomeres (human umbilical vein endothelial cells, passage 5). A standard curve was generated from a serially diluted reference DNA sample of human gDNA (Roche #11,691,112,001) for each of the qPCR runs to ensure primer and qPCR efficiency.

The final relative telomere length (T/S ratio) for each sample was the ratio of Telomere (T) and 36B4 (S) amplification which was calculated based on the qPCR efficiency and relative to the inter-run calibrators, as previously described [9].

The entire telomere length analysis was performed in a blinded, anonymized, and randomized experimental setup.

### Statistical analysis

IBM SPSS Statistics (IBM, New York, NY), version 29.0, was used for the statistical analyses. For the analysis, a mixed linear model was calculated using a restricted estimated maximum likelihood approach to compare telomere length between different time points. The mixed linear model in SPSS can in general be used to develop different variance analysis models. It can deal with a lot of variables and covariates. The level of significance was set at *p* ≤ 0.05 throughout the analysis while correcting for multiple comparisons for the post hoc *t*-tests (Bonferroni). The telomere lengths were defined as the dependent variable and fixed factors were the time points of measurement. Age, sex, smoking behavior, BMI, nicotine consumption, and ethanol consumption were used as covariates. The covariates were used to exclude whether they have a significant influence on alterations in telomere length.

For correlation analyses of telomere lengths and BMI, nicotine consumption, alcohol consumption, and the WHOQOL-BREF Social Score, we used a bivariate correlation in SPSS.

## Results

### Demography

According to the study design, only older participants were included in the study with a mean age of 68.9 (± 2.9) years in the piano group and 69.5 (± 4) years in the control group. Groups showed a comparable distribution of sex with approximately equal portions of females and males (Table 2). As a marker for the general fitness and health status of the study participants, the PASE (Physical Activity Scale for the elderly) has been implemented in the table, showing an even higher activity and health score as compared to the norm values in this age class [10], which is 112 to 144.

**Table 2:** Demographics of the study cohort

	Piano group	Control group
Mean age ± SD (years)	68.9 ± 2.9	69.5 ± 4
Min./Max	64/76	62/78
*N*	29	26
Female/male (%)	44.8/55.2	38.5/61.5
PASE (Physical Activity Scale for the Elderly)	157 ± 12.4	167.8 ± 18

### Health conditions

Study participants were also asked for general health conditions, comprising the most important disease complexes like cardiovascular problems, hypertonia, neurological problems, Parkinson disease, diabetes, arthrosis, arthritis, rheuma, and diseases of the thyroidal gland. Table 3 shows the number of study participants included in the analyses of telomere lengths and their affection by chronic diseases, revealing an overall good health in the participants. Only a small proportion of the participants suffered from the diseases surveyed. This proportion was only higher in case of hypertonia and arthrosis. Therefore, to exclude an effect of these two health conditions on the development in telomere length, we also made a correlation analyses with the telomere lengths at the three different timepoints but found no significant correlation.

**Table 3:** Health conditions of study participants

Disease	Number of participants	Number of participants affected by the disease	% of affected participants in relation to all participants
Cardiovascular	55	4	7.3
Hypertonia	55	18	32.7
Neurological	55	3	5.5
Parkinson	55	0	0.0
Diabetes	55	0	0.0
Arthrosis	55	18	32.7
Arthritis	55	2	3.6
Rheuma	55	4	7.3
Thyroid	55	4	7.3

### BMI, consumption of nicotine and alcohol

The mean BMI in our study cohort was 24.5 ± 0.37 (min, 19; max, 34.8; *n* = 55). Assessment of nicotine and alcohol consumption revealed 8.44 ± 1.75 years of smoking (min, 0; max, 45 years; *n* = 55) and 27.15 ± 3.21 years (min, 0; max, 60 years; *n* = 55) of occasional alcohol consumption.

### Psychometric measurements: course of the WHOQOL-BREF Social Score

The WHOQOL-BREF Social Score showed a very slight and insignificant decline from 75.3 ± 2.0 at baseline (T0) to 74.2 ± 2.1 at T1. From T1 to T2, there was a bit more pronounced diminution from 74.2 ± 2.1 to 72.5 ± 2.3, which was also not significant (Table 4).

**Table 4:** WHOQOL-BREF subscores of study participants on T0, T1, and T2

	T0 Mean ± SEM (*n*)	T1 Mean ± SEM (*n*)	T2 Mean ± SEM (*n*)
WHOQOL-BREF Social Score	75.3 ± 2.0 (55)	74.2 ± 2.1 (55)	72.5 ± 2.3 (55)

### Measurement of telomere lengths

**Fig. 1:**
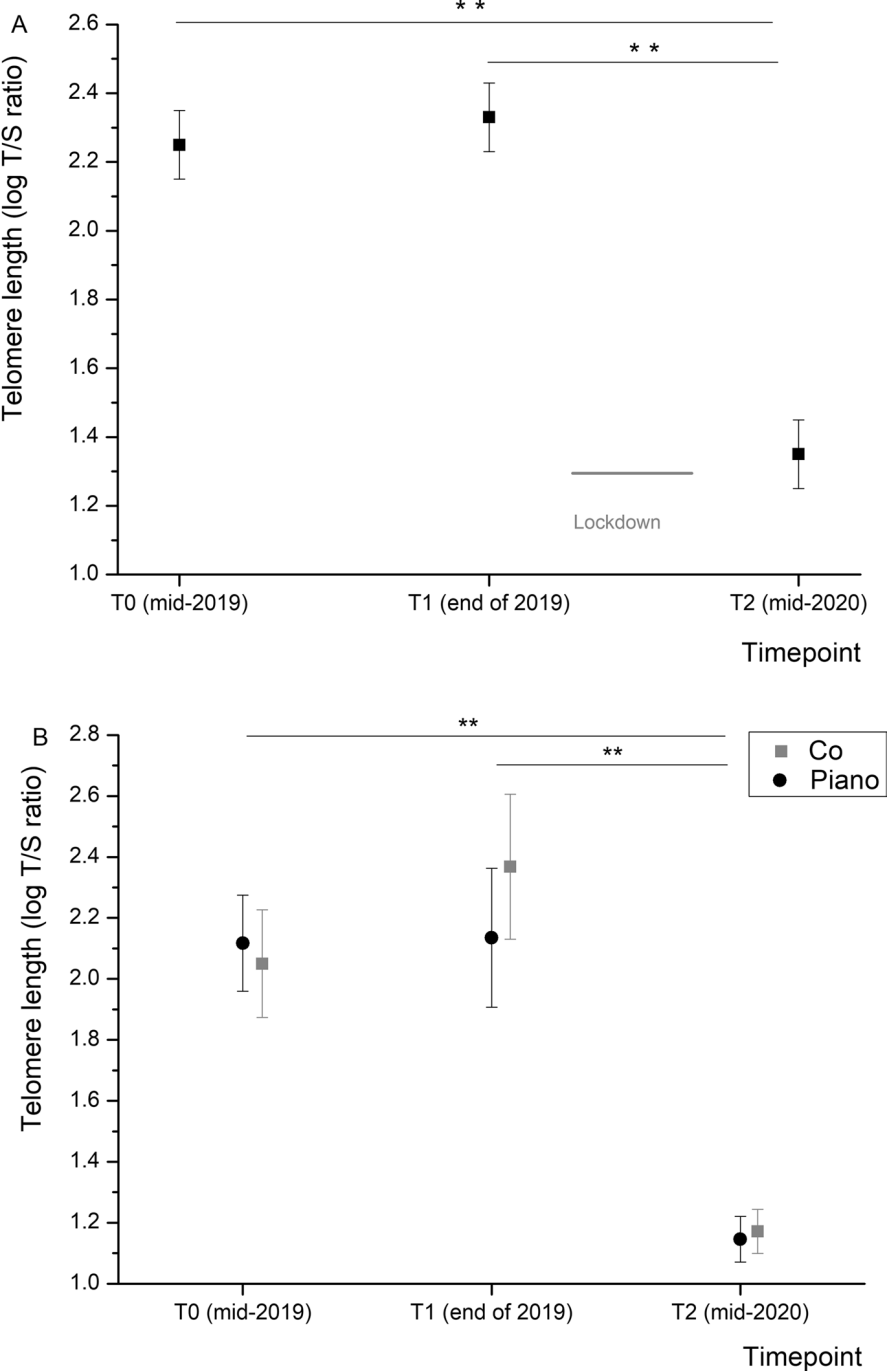
**A** Changes in telomere length over time (mean ± S.E.M.) showing a highly significant difference between T1 and T2 (*p* < 0.01) as well as T0 and T2 (*p* < 0.01), respectively (*n* = 55). **B** Separate representation of the results of the two groups. As to be seen, there were no differences in telomere length between the piano and the control group

The telomere length measurements revealed no significant differences between time points T0 (mid of 2019) and T1 (end of 2019). There were also no differences in telomere length between the piano and the control group (Fig. 1B). There was a large and highly significant drop in telomere length from time point T1 to T2 (mid of 2020) in both groups. As there were no significant differences between groups, and the focus of the current short report is the effect of the lockdown on telomere length, the data from the piano and control were grouped. In the combination of the two groups, the average telomere length was 2.25 (± 0.1 S.E.M.) at T0, 2.3 (± 0.1) at T1, and 1.35 (± 0.1) at T2 with a highly significant difference between T1 and T2 (*p* < 0.01) and T0 and T2 (*p* < 0.01) (Fig. 1A). For the sake of completeness, we also evaluated the excluded study participants with a T2 before the lockdown. The respective results revealed only a small drop in relative telomere length (the difference between T1 and T2 was 0.4), but this did not reach significance (*p* > 0.05). As mentioned in the methods part, age, sex, BMI, and nicotine and ethanol consumption were set as covariates in the analyses in order to correct for any potential effects of these factors on our findings. Table 5 shows the corresponding *F*-values and significance levels of these covariates.

**Table 5:** F-values of covariates

	Counter degrees of freedom	Denominator degrees of freedom	*F*-value	Significance
BMI	1	147	1.6	0.202
Nicotine consumption	1	147	0.03	0.871
Ethanol consumption	1	147	0.09	0.762
Sex	1	147	2.9	0.092
Age	1	147	1.2	0.276

### Correlation of BMI, nicotine consumption, and alcohol consumption with telomere lengths

There were no significant correlations of BMI, nicotine, or alcohol consumption with the telomere lengths at any of the timepoints. For BMI, the correlation coefficients ranged from 0.02 to 0.03 (*p* = 0.8 each), for nicotine consumption values were 0.05 (*p* = 0.6), and for ethanol consumption values spanned from 0.07 to 0.08 (*p* = 0.4 to 0.5) for the different time points. This was also confirmed by our findings from the mixed linear model analyses which did not show any significant influence of these three parameters as covariates on the telomere lengths.

### Correlation of WHOQOL-BREF Social Score and telomere lengths

The correlation of the telomere length decline with the WHOQOL-BREF Social Score decline narrowly missed reaching the significance level as the Pearson correlation coefficient was 0.24 (*p* = 0.07).

### COVID-19 infection rate

None of the study participants had undergone an infection with COVID-19 (coronavirus SARS-CoV-2) at or before the time points of our study assessment.

## Discussion

When we analyzed our data for the first time, we were surprised by the very strong drop in telomere length from time point T1 (end of 2019) to T2 (mid of 2020), which could not be correlated to our study design. First, we ruled out any systematic mistakes during blood withdrawal and laboratory procedures by carefully revisiting all parameters. The blood samples were always taken by the same persons in Hannover and Geneva without any change in the standard operating procedure between the different rounds of sample collections. During the laboratory measurements, the samples were randomized to avoid plate and qPCR run effects, i.e., each qPCR run contained samples from all time points, eliminating the measurement bias that may occur if the different time points were exclusively run on separate plates.

The decline was higher than expected under normal conditions in the physiological process of aging [11, 12]. This can also be depicted from the difference between time points T0 (mid-2019) and T1 (end of 2019) before the lockdown, which comparatively spans also a timeframe of 6 months, as a kind of control where there was almost no change in telomere length. Therefore, it was a real stroke of luck that we also had the data from this comparative period.

The strong decline in telomere length did not correlate with any physical issues of the study participants. In general, the cohort was in good general health status as shown in Table 3. Further support regarding this point also came from the PASE scores, showing our study participants to have even higher scores of physical activity than the reference values of the test for their age class [10].

Furthermore, the BMI of our study participants was in the normal range for adults and older persons and it did also not correlate with the telomere length. Concerning the consumption of nicotine and alcohol, there was a kind of heterogeneity in the study cohort as some participants did neither consume nicotine nor alcohol whereas others did occasionally. However, there was no significant correlation with telomere length, neither indirectly as covariates in the mixed linear model nor in the direct bivariate correlation calculation.

However, anyway, it was not very probable that more static and chronic parameters like the discussed ones could explain the sudden drop in telomere length in the second half-year whereas it was stable in the first observational-half-year.

Considering social life, we found a slight and insignificant decrease in the WHOQOL-BREF Social Score, which was more pronounced between T1 and T2. However, the correlation of the decline in telomere lengths with the decline in this psychometric measurement narrowly missed the level of significance. It could be hypothesized that we missed the level of significance due to low n-numbers. However, this trend could give a hint that the changes in telomere length could at least partially be associated with alterations in the social interactions of the study participants. When thinking about the time and environmental factors during the period when samples were acquired, it became clear, that there were profound social intrusions into everyone’s life due to the first lockdown in the context of Corona-policies.

It is known that lockdown-associated deprivations led to severe psychosocial risks [13] like increased domestic violence [14] and critical psychological distress in 50% of the population with severe effects in even 20% [15, 16] worldwide and in all age groups. Some authors predicted already in 2020 kind of a “parallel pandemic” of acute stress disorders and other psychological problems [15, 17].

Fittingly, the group of Luxton et al. [18] made a similar observation in the context of telomerase activity and telomere length measurements performed for astronauts who went on a space mission before and after the spaceflight. The reduction in telomere length in such a short span of time was attributed to chronic oxidative stress experienced by the astronauts. Furthermore, participants of space missions are locked in a small space and are restricted in their freedom of movement. Equivalently, European and Swiss citizens also experienced high levels of stress as a consequence of the implementation of Corona-lockdown policies. Considering the temporal and environmental factors that prevailed during the period in which the samples were acquired reveals a profound impact of the initial lockdown on the lives of all individuals.

To date, telomere length has been rather recognized as a biomarker to predict the severity of the course of SARS-CoV-2 infections as different studies found an increased risk for a severe course of infections with short telomere lengths [19] and COVID-19 survivors with severe decline in lung function had significantly shorter telomere lengths compared to the patients with a mild effect on lung function after SARS-CoV-2 infection [7]. Accordingly, it was known from the beginning that older people were at significantly higher risk of severe courses of SARS-CoV-2 infections compared to younger people [20].

To our knowledge, there is only one other study so far, which among other parameters includes some information concerning the development of telomere length during lockdowns, showing only an insignificant decrease of − 0.6 to − 0.87 after lockdown [21]. However, the telomere length in that study had been estimated with a special algorithm from epigenome-wide methylation data. It is known that the reliability of such data strongly depends on the sequencing coverage [22]. In the current study, we used a well-established approach for the relative measurement of telomere lengths. These methodological differences as well as a slightly different study design concerning time points when data were collected, for example, could in part explain why we found a more pronounced and significant decline in telomere length (approximately − 1.0).

It could be argued that the reduction of telomere length we observed could also result from an infection with SARS-CoV-2, a coherence that has been shown by Victor et al. [23]. However, there are also studies that could not find any significant association between COVID-19 and LTL [24, 25]. Anyways, as already mentioned before, the participants in the current study did not experience any infections with SARS-CoV-2 during the period of observation. This is also in line with statistics showing the incidence rates of Corona in the year 2020. There were almost no infections until March 2020, when the first slight peak in infections occurred as indicated by the number of infections per 100,000 citizens for 14-day intervals, revealing infection numbers < 100 on average for all federal states in Germany [26]. In April, infection rates almost returned to zero and the next peak (with infection numbers of 300 to 400) occurred as late as in August 2020 [26] when our data and blood assessment of T2 was already finished. In Switzerland, the situation was comparable.

## Limitations

As the study did originally not aim to investigate the effects of SARS-CoV-2 measures on telomere length, we only had psychometric data, which were chosen to answer questions concerning neuroplasticity. Therefore, we only had a few measures for social interaction.

Furthermore, due to the study design, we can only provide information about the development of telomere length in older people but not in the broader population.

Due to the study design offering piano lessons, it might be conceivable that we have tended to attract people with a higher level of education and higher socio-economic status. Therefore, it is possible that this also created a bias in our psychometric measurements because it is known that people with a lower socio-economic status suffered more from the lockdowns. However, this again stresses the relevance of our finding as it might have been even stronger in a less protected subset of the broader population.

Unfortunately, there was no data regarding the stress hormone axes. This could have given interesting insights and a more comprehensive picture.

Furthermore, we only had a relatively small sample size (*n* = 55). Therefore, results should rather be considered preliminary. The low n-number might also be an explanation for why the level of significance was narrowly missed in the development of the WHOQOL-BREF and the corresponding correlation.

## Summary and conclusion

Although the study originally had a different aim, this important incidental and consequential finding of a high loss in telomere length during the first lockdown as a measure of Corona policy in Germany and Switzerland is noteworthy in our opinion. Our study supports the ongoing demand for systematic processing and re-evaluation of measures as our finding points to the adverse effects of lockdowns, even in the age class that was meant to be protected the most by the measure. Considering the cited hazardous effects of lockdowns on persons younger than 50 years, especially children, at almost no risk for severe infections, there is a clear need to learn from this and alter strategies for the future.

## Outlook and future perspectives

During situations like the COVID-19-pandemic, there should be a more comprehensive data acquisition especially from the official side in order to better access the situation and plausibility of measures. Longitudinal studies investigating the long-term effects of lockdowns on telomere length and cognitive function would be desirable to provide deeper insights into the biological impact of lockdowns.

## Data Availability

Data are available from the corresponding author upon request via Jahn.Kirsten@mh-hannover.de.

## Abbreviations

T0: Timepoint 0
T1: Timepoint 1
T2: Timepoint 2
DNA: Deoxyribonucleic acid
PCR: Polymerase chain reaction
LTL: Leucocyte telomere length
FW: Forward
REV: Reverse
S.E.M.: Standard error of the mean
Suppl.: Supplemental
Fig.: Figure
